# Co‐Design of Strategies to Support the Implementation of a Paediatric Risk‐Reduction Pathway for Familial Hypercholesterolaemia

**DOI:** 10.1111/jpc.70447

**Published:** 2026-05-31

**Authors:** Mitchell Sarkies, Shubha Srinivasan, Ari Horton, Jacquie Garton‐Smith, Gerald F. Watts, Christine Tawtel, Jenny Della‐Vedova, Kristen J. Nowak, Jing Pang, Andrew Martin

**Affiliations:** ^1^ Sydney School of Health Sciences, Faculty of Medicine and Health The University of Sydney Sydney New South Wales Australia; ^2^ Implementation Science Academy Sydney Health Partners Sydney New South Wales Australia; ^3^ Institute of Endocrinology and Diabetes The Children's Hospital at Westmead Sydney New South Wales Australia; ^4^ Discipline of Child and Adolescent Health, Faculty of Medicine and Health University of Sydney Sydney New South Wales Australia; ^5^ Victorian Heart Institute Monash University Melbourne Victoria Australia; ^6^ Victorian Heart Hospital Melbourne Victoria Australia; ^7^ Paediatric Cardiology Monash Children's Hospital, Monash Health Melbourne Victoria Australia; ^8^ School of Public Health and Preventative Medicine Monash University Melbourne Victoria Australia; ^9^ Department of Paediatrics Monash University Melbourne Victoria Australia; ^10^ Health Networks, Clinical Excellence Division Department of Health Western Australia East Perth Western Australia Australia; ^11^ Royal Perth Bentley Group East Metropolitan Health Service Perth Western Australia Australia; ^12^ Medical School University of Western Australia Perth Western Australia Australia; ^13^ Lipid Disorders Clinic, Cardiometabolic Service, Departments of Cardiology and Internal Medicine Royal Perth Hospital Perth Western Australia Australia; ^14^ FH Australia Perth Western Australia Australia; ^15^ Department General Paediatrics Perth Children's Hospital Perth Western Australia Australia; ^16^ Division of Paediatrics, Faculty of Health and Medical Sciences University of Western Australia Perth Western Australia Australia; ^17^ Office of Population Health Genomics, Public and Aboriginal Health Division Western Australian Department of Health East Perth Western Australia Australia; ^18^ School of Biomedical Science, Faculty of Health and Medical Sciences University of Western Australia Perth Western Australia Australia

## Abstract

**Objectives:**

Familial hypercholesterolaemia (FH) affects 1 in 311 people. Left untreated, it elevates LDL‐cholesterol from early life and drives premature atherosclerotic cardiovascular disease. Despite paediatric guidelines for early lipid‐lowering, detection and management in Australia remain suboptimal. Shared‐care models across general practice, paediatrics, and FH‐specialist services are promising but under‐implemented. We co‐developed implementation strategies to support a paediatric FH risk‐reduction pathway.

**Methods:**

Two qualitative focus‐group workshops with clinical, policy and advocacy stakeholders were conducted. Transcripts underwent inductive and deductive thematic analysis. Barriers and facilitators were mapped to the Consolidated Framework for Implementation Research and strategies to the Expert Recommendations for Implementing Change, with structured specification of actor, action, target, temporality and dose.

**Results:**

Barriers included unclear responsibility across settings, limited clinician confidence, constrained access to specialist dietary advice and treatment non‐adherence. Facilitators included patient/family empowerment, existing financial reimbursement and engaged clinical champions. Nine strategies emerged: (1) treatment‐guidance letters; (2) coordination and phone support; (3) individualised patient passports; (4) financial reimbursement; (5) diet and lifestyle resources; (6) patient and family engagement; (7) decision support systems; (8) audit and feedback; and (9) development of clinical champions.

**Conclusions:**

A co‐designed suite of nine strategies could enable shared‐care for paediatric FH in Australia. This structured, replicable package is adaptable across jurisdictions and can support earlier detection and improved management. Evaluation of effectiveness and scalability in routine care is warranted, with potential relevance to other paediatric lipid disorders.

## Brief Points

- What is already known on this topic?
  ○ Familial hypercholesterolaemia is common and raises LDL‐cholesterol from childhood, causing premature ASCVD unless treated early.
  ○ In Australia, most affected children remain undetected and few reach LDL‐cholesterol targets despite recommendations to start lipid‐lowering therapy from ages 8 to 10.
  ○ Shared‐care between FH‐specialists, paediatricians, and general practitioners is generally advocated but under‐implemented.
- What this paper adds?
  ○ This study co‐designed nine actionable strategies to implement a paediatric FH risk‐reduction pathway.
  ○ It delineates roles for tertiary clinics, community paediatrics and general practice, allowing flexibility to different jurisdiction needs.
  ○ Our replicable shared‐care model could enable care to be scaled beyond centralised lipid clinics.

## 1 Introductions

Familial hypercholesterolaemia (FH) is one of the most common autosomal monogenic conditions affecting 1 in 311 people worldwide [1]. Children have a 50% chance of inheriting FH from an affected parent, which leads to significantly elevated low‐density lipoprotein (LDL) cholesterol levels from birth or early childhood. If left untreated, FH often causes premature atherosclerotic cardiovascular disease (ASCVD) and shortens life expectancy by 20 years for men and 30 years for women [2]. For children with untreated homozygous FH, life expectancy may be under 30 years [3]. Detecting and treating FH in childhood best prevents premature ASCVD and supports a normal lifespan [4].

It is estimated there are up to 20 000 children with FH in Australia [5]. However, less than 10% have been detected [5] and only 20% achieve guideline‐recommended LDL‐cholesterol targets, usually not until adulthood or after their first cardiac event [6]. Several clinical practice guideline updates [7, 8] and implementation roadmaps [9] have been published to promote early initiation of lipid‐lowering treatment from age 8 to 10 years in both males and females [8]; however, care gaps persist [10, 11].

Shared‐care models between paediatric lipid specialists, general paediatricians and family general practitioners (GPs) have been proposed to integrate multidisciplinary health services [8, 9, 12, 13]. However, implementation has been hampered by barriers such as hesitation amongst paediatricians and GPs to initiate children on lipid‐lowering treatments [14], despite evidence of safety and endorsement internationally [15]. The practical implementation of a shared‐care model for the management of FH in childhood requires development of risk‐reduction pathways that are co‐designed with end‐users, clearly delineate roles, responsibilities, timing, and settings for different aspects of care, supported by strategies for implementation.

The aim of this study was to co‐develop potential strategies for the implementation of a paediatric risk‐reduction pathway for FH in Australia.

## 2 Methods

### 2.1 Design, Setting and Participants

We conducted two face‐to‐face focus groups to co‐design implementation strategies for a paediatric FH risk‐reduction pathway (see Appendix S1 researcher characteristics). The first 2‐h state workshop was hosted on 7 August 2024 in Perth, Australia. It was co‐facilitated by an implementation scientist (MS) and paediatrician (AM) as part of the FH in Kids project which aims to identify children with FH in WA, Australia. The second 1.5‐h workshop was facilitated by an implementation scientist (MS) and was organised as the final session of the 2024 FH Summit on 27 October 2024. The FH Summit, hosted by the FH Australasia Network, brought together a panel of Australian experts to provide updates on several areas of the management of FH. Field notes were taken and both workshops were audio recorded and transcribed verbatim (see Appendix S2 facilitator guide). The first workshop included a purposive sample of Western Australian FH care representatives identified and invited by AM from their existing professional network, referred to as the 10‐Ps used in previous research [9, 13]: patients, GPs, physicians, paediatrics, precision medicine, pathology, nurse practitioner, psychology and dietetics and policy makers. The second workshop at the FH Summit included a similar sample of representatives invited by the event hosts from an existing professional network (FH Australasia Network). All of the 10‐Ps were represented in the first workshop, eight of whom also attended the second workshop. The second workshop did not include a psychologist or policy maker.

The authors declare that the research presented in this manuscript adheres to the ethical principles outlined by Perth Children's Hospital, Child and Adolescent Health Service (quality improvement activity number: 54535). All procedures involving human participants were conducted in accordance with the ethical standards of the National Statement on Ethical Conduct in Human Research and the Declaration of Helsinki (1964), as revised in 2013. This study has been reported according to the Standards for Reporting Qualitative Research guideline (Appendix S3).

### 2.2 Data Analysis

Transcripts and field notes were read in full, and a two‐stage analysis approach was followed. Stage 1 was an inductive and deductive thematic analysis to identify and categorise barriers and facilitators to the implementation of a paediatric FH risk‐reduction pathway. Implementation barriers and facilitators were inductively coded using axial coding to develop a hierarchical structure of codes, before deductive mapping of barriers and facilitators to the Consolidated Framework for Implementation Research (CFIR) [16]. Stage 2 was also an inductive and deductive thematic process, whereby strategies to support the implementation of a paediatric FH risk‐reduction pathway were inductively coded and then deductively mapped to the Expert Recommendations for Implementation Change (ERIC) taxonomy [17]. Each strategy was described according to guidance on specifying and reporting implementation strategies, reporting: (1) the actor (who), (2) action (what), (3) action target (who is impacted), (4) temporality (how often) and (5) dose (how much) (Table 1). Analysis was undertaken by a single researcher (MS) and data were managed and coded using QSR NVivo software. Rigour and trustworthiness were maintained through triangulation with field notes, memo writing and reflexive consideration of the researcher's disciplinary background and assumptions throughout data collection and analysis [19].

**TABLE 1:** Domains for specifying and reporting of implementation strategies.

Domains	Definition
Name it	Name the strategy, preferably using language that is consistent with existing literature (e.g., ERIC compilation).
Define it	Define the implementation strategy and any discrete components operationally.
Specify it	
The actor	Identify who enacts the strategy (e.g., administrators, payers, providers, patients/consumers, advocates, etc.).
The action	Use active verb statements to specify the specific actions, steps, or processes that need to be enacted.
Action target	Specify targets according to conceptual models of implementation Identify unit of analysis for measuring implementation outcomes.

*Note: Source:* Reproduced from Proctor et al. [18] with permission by BioMed Central Ltd.

## 3 Results

**FIGURE 1:**
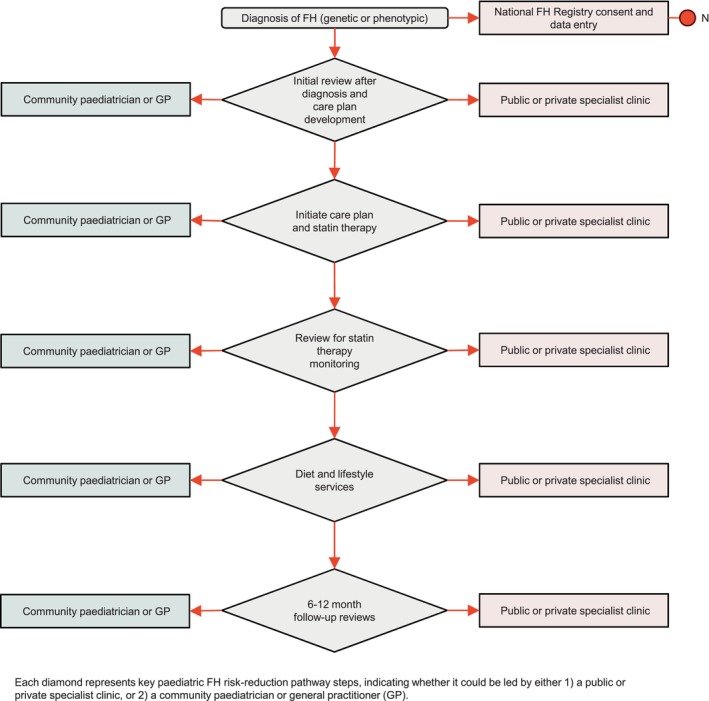
Process map of key paediatric FH risk reduction pathway steps and organisational configurations for implementation.

Two focus group workshops were held: a state‐level workshop with *n* = 20 participants, followed by a national workshop with *n* = 41 participants (Table 2). Key paediatric FH risk‐reduction pathway steps and organisational configurations for implementation were identified and summarised in Figure 1. Each step is summarised, indicating whether it could be led by (1) a public or private FH specialist clinic, or (2) a community paediatrician or GP. Six barriers (Table 3) and three facilitators (Table 4) to the implementation of an FH paediatric risk‐reduction pathway were identified and mapped according to the CFIR domains and constructs [16].

**TABLE 2:** Workshop participant demographics.

	State level	National level
General practitioner	4 (20%)	5 (12%)
Patient	4 (20%)	2 (5%)
Precision medicine	1 (5%)	3 (7%)
Nurse practitioner	2 (10%)	1 (2%)
Paediatrician	1 (5%)	9 (22%)
Pathologist	1 (5%)	4 (10%)
Physician	2 (10%)	12 (29%)
Psychologist	1 (5%)	0 (0%)
Policy maker	2 (10%)	0 (0%)
Dietitian practitioner	1 (5%)	2 (5%)
Implementation researcher	1 (5%)	3 (7%)
Total	20	41

**TABLE 3:** Barriers to the implementation of a paediatric risk reduction pathway for FH.

Implementation barriers	Example quote	CFIR domains and constructs
Blurred lines of responsibility for FH management	“A shared‐care model is about a GP starting care and calling in a non‐GP to assist them in achieving best outcomes” [FH Summit GP 1]	Innovation Complexity Adaptability
		Inner setting Compatibility
		Implementation process Teaming
Lack of access to FH specialist guidance for management	“I think we need to be a bit more creative and innovative of how we access non‐GP specialists” [FH Summit Paediatrician 1]	Inner setting Available resources Work infrastructure Relative priority
		Outer setting Partnerships and connections Financing
Limited clinician knowledge and skills in FH	“I think a key issue, really, is the education of clinicians across the board” [FH Summit Cardiologist 1]	Inner setting Access to knowledge and information
Treatment non‐adherence in FH	“I'm thinking, from [an] early age, and for many of these families it's that's kind of the last thing on their radar” [WA Meeting Clinical Psychologist 1]	Implementation processes Assessing needs Engaging with innovation recipients
Lack of access to dietitian advice and programs for FH	“the cost to see a dietician, even with a rebate, still at 100 plus dollars every time. And that's really not equitable for lots of our families” [WA Meeting Dietitian 1] “I need access to a dietitian. From my perspective, that's what the shared care model means; that as a GP, I can access that service in a slightly, hopefully more flexible way than I can in the community for this high‐level service.” [FH Summit GP 2]	Innovation Adaptability Cost
		Inner setting Available resources
General paediatrician and GP readiness for managing FH	“If I walk around (the hospital), none of the paediatricians would be comfortable prescribing statins, none of them, because they don't prescribe them in any other condition” [WA Meeting Paediatrician 2] “GPs really didn't feel equipped for the most part to have to counsel children and their families” [WA Meeting Physician 1]	Individuals Capability of innovation deliverers

**TABLE 4:** Facilitators to the implementation of a paediatric risk reduction pathway for FH.

Implementation facilitators	Example quote	CFIR domains and constructs
FH patient and family empowerment	“some of that might be about empowering families and making sure that they are aware and knowledgeable and able to reach back in personally to a tertiary centre, or wherever their lipid clinic is, if they feel they're not getting the treatment that they want” [FH Summit Patient Advocate 1]	Implementation process Engaging innovation recipients
Financial reimbursement for chronic disease management of FH	“because it's a chronic condition, we can do care plans once the diagnosis is made. We can review them every three or four months, and you can get remunerated. And it's a good incentive. It keeps you focused on it and it keeps you focused on the outcomes. You bring them back every four months and do it. And it is very rewarding” [FH Summit GP 3] “this has to be funded from somewhere. People say you could just incorporate it into what you're doing in general practice. But, there's an opportunity cost, there isn't it. If you're doing more of this, there's something else that you're not doing.” [FH Summit Physician 1]	Outer setting Financing
FH champions and boundary spanners	“GPs need to own it… Stakeholder involvement is important. And what do I mean by that? You know, champions. And what do I mean by that? People who are good boundary spanners… But also, people who are actually doing it” [FH Summit Physician 1]	Individuals Opinion leaders

### 3.1 Barriers to Implementing a Paediatric Risk‐Reduction Pathway for FH

Barriers to implementing a paediatric FH risk‐reduction pathway included unclear responsibilities between general paediatricians, GPs, and FH specialists, leading to confusion after referrals. Limited access to FH specialist advice was also a challenge, especially in rural or under‐resourced areas. Clinician knowledge gaps and discomfort with initiating treatment contributed to delays in care. Treatment non‐adherence was common, particularly amongst families facing instability or financial hardship. Access to dietitian services was limited by cost, and the low prevalence of diagnosed FH cases in practice reduced clinician readiness and confidence to initiate and escalate treatment (Table 3).

### 3.2 Facilitators to the Implementation of a Paediatric Risk‐Reduction Pathway for FH

Facilitators identified to support implementation of a paediatric FH risk‐reduction pathway included support for a shared‐care model with clearly defined roles and referral timelines, use of case conferencing and care planning to manage bottlenecks, and flexible access to FH specialist input through virtual care. Stakeholders also emphasised the importance of timely and specific communication, integration of genetic counselling and allied health services, and investment in telehealth infrastructure and funding mechanisms to enable equitable access (Table 4).

### 3.3 Implementation Strategies to Support a Paediatric Risk‐Reduction Pathway for FH

Nine strategies to support the implementation of a paediatric risk‐reduction pathway for FH were identified (Table 5). The description of each strategy was standardised to specify the reporting of implementation strategies to allow for their operationalisation and replication [19]. Each strategy was described according to the actor (who), action (what), action target (who is impacted), temporality (how often), and dose (how much).

1. Treatment guidance letter (distribute educational materials): It was suggested that a letter with guidance on post‐test counselling, initiating statins, treatment targets, a visual illustration of early treatment benefits, and further resources be provided after return of a positive FH genetic test result. The letter could be templated and provided by the public or private FH specialist clinic in either an electronic or hard‐copy format to the patient and their family, as well as their general paediatrician or GP.
2. Coordination and phone support (facilitation): A coordination model involving designated phone support was proposed to facilitate communication across care settings. Nursing staff based in public or private FH specialist clinics, general paediatrics, or general practice would manage a point‐of‐care support, offer advice, and coordinate care between providers and families. This strategy would be available on an as‐needed basis.
3. Personalised patient passport (prepare patients to be active participants): An individualised patient passport was identified as a strategy to promote care continuity and patient empowerment. It could be developed by health departments or public and private FH specialist clinics following a confirmed FH diagnosis. This tool would be digitised and include summarised medical and family history and be used by families during appointments with new healthcare providers.
4. Financial reimbursement for managing FH (access new funding): Participants identified financial reimbursement as a key enabler of sustained, high‐quality care for paediatric FH. GPs managing a paediatric patient with a confirmed FH diagnosis could utilise existing Medicare Benefits Schedule (MBS) Items for chronic disease management plans, allowing for remuneration for both initial care planning and regular follow‐up (every 3–4 months).
5. Resources for diet and lifestyle (distribute and develop educational materials): High‐quality dietary and lifestyle resources were considered integral to facilitate effective FH management. These materials would be developed and promoted by public or private FH specialist clinics and health consumer advocacy organisations to be distributed after diagnosis of FH. Resources should be culturally appropriate, age‐appropriate, and suitable for a range of literacy levels, with the goal of supporting patients and their families with long‐term lifestyle modifications and treatment adherence.
6. FH patient and family engagement (involve patients and family members): It was viewed that all stakeholders should facilitate FH patient and family engagement in healthcare decisions. Clear communication, educational tools, and ongoing support available as needed for patients and families would empower them to recognise when treatment goals are not being met and to proactively seek further care if needed.
7. Decision support systems (remind clinicians): The development of clinical decision‐support systems was proposed to aid health professionals in recalling key information and prompting appropriate care processes. These systems would be integrated within existing clinical software tools and serve to remind general paediatricians and GPs about follow‐up schedules, treatment thresholds, and guideline‐based prescribing.
8. Audit and feedback quality assurance cycle (audit and provide feedback): An audit and feedback cycle, leveraging infrastructure such as the National FH Registry, was identified as a strategy to drive ongoing quality improvement. Clinical performance data would be benchmarked against guideline recommendations and shared with contributing clinicians to support quality assurance and professional development. The frequency of feedback would be determined by the health professional, aligning with their preferred approach to continuous improvement.
9. Identify and prepare clinical champions (identify and prepare champions): Identifying and equipping clinical champions was recognised as a key driver of healthcare practice change. These individuals would be ideally general paediatricians and GPs with experience in paediatric FH management, playing a leadership role in overcoming resistance to implementation, supporting peer learning, and modelling best practice across healthcare settings. Public or private FH specialist clinics, general paediatrics, and general practice, health consumer advocacy organisations, university medical schools, and specialist medical colleges would be responsible for identifying and preparing these champions to support local adoption efforts.

**TABLE 5:** Implementation strategies for a paediatric FH risk reduction pathway according to the actor (who), action (what), action target (who is impacted), temporality (how often), and dose (how much).

Strategy name	Treatment guidance letter (distribute educational materials)
Strategy definition	Letter with guidance on post‐test counselling, initiating statins, treatment targets, a visual illustration of early treatment benefits, and further resources
Actor	Public or private FH specialist clinics
Action	Provide templated electronic or hard‐copy letter that can be individualised
Action target	General paediatricians, GPs and FH patients and their families
Temporality and dose	After return of FH genetic test results and each subsequent review (if required)
Strategy name	Coordination and phone support (facilitation)
Strategy definition	Targeted point‐of‐care support that includes a phone number to call or e‐mail address for advice
Actor	Public or private FH specialist clinics, general paediatrics and general practice
Action	Nursing staff coordinate communication and care between primary and tertiary settings
Action target	General paediatricians, GPs and FH patients and their families
Temporality and dose	Available as needed
Strategy name	Individualised patient passport (prepare patients to be active participants)
Strategy definition	Create a patient passport that includes a summarised medical and family history that is digitised and able to be included in the health record (e.g., My Health Record)
Actor	Public or private FH specialist clinics and general practice
Action	Create an individualised patient passport that patients and families can bring to appointments with new health professionals
Action target	General paediatricians, GPs and FH patients and their families
Temporality and dose	After confirmation of FH diagnosis and updated when circumstances change
Strategy name	Financial reimbursement for managing FH (access new funding)
Strategy definition	Financial reimbursement to incentivise the appropriate management of FH in general practice, reimburse the cost to patients of accessing allied health services, support treatment adherence and handover between care settings and transition to adult services
Actor	General practices with a confirmed paediatric FH patient
Action	Utilise the existing MBS Item for Chronic Disease Management plans
Action target	FH patients and their families
Temporality and dose	Once every 12 months and can review once every 3 months
Strategy name	Resources for diet and lifestyle advice (distribute and develop educational materials)
Strategy definition	Develop and promote high‐quality resources for diet and lifestyle management
Actor	Public or private FH specialist clinics and health consumer advocacy organisations
Action	Distribute existing and create new resources
Action target	General paediatricians, GPs and FH patients and their families
Temporality and dose	Once after return of a positive FH genetic test result and available as needed thereafter
Strategy name	FH patient and family engagement (involve patients and family members)
Strategy definition	Facilitate FH patient and family engagement in healthcare decisions and processes.
Actor	Public or private FH specialist clinics, general paediatrics and general practice, health consumer advocacy organisations
Action	Empower patients with the information to be engaged in their care decisions and processes
Action target	FH patients and their families
Temporality and dose	As needed
Strategy name	Decision support systems (remind clinicians)
Strategy definition	Develop and implement decision support systems to assist health professionals to recall information and prompt care processes
Actor	Academic partners, practice software companies, Primary Health Networks
Action	Integrate decision support systems within existing tools used by health professionals
Action target	General paediatricians, GPs and FH patients and their families
Temporality and dose	As needed
Strategy name	Audit and feedback quality assurance cycle (audit and provide feedback)
Strategy definition	Establish an ongoing audit and feedback process using existing infrastructure such as the National FH Registry or new infrastructure, whereby clinical performance data is benchmarked against guideline recommendation and provided to health professionals to encourage quality assurance and professional development activities.
Actor	National FH Registry, Primary Health Networks
Action	Systematically collect and report FH clinical performance data to health professionals.
Action target	Registry clinician contributors
Temporality and dose	At intervals determined by the individual health professional.
Strategy name	Identify and prepare clinical champions (identify and prepare champions)
Strategy definition	Identify and prepare health professions with an interest in providing care for FH patients who are dedicated to supporting implementation and overcoming resistance to clinical practice change. Ideally this would include general paediatricians and GPs whose role spans the tertiary and primary care settings.
Actor	Public or private FH specialist clinics, general paediatrics and general practice, health consumer advocacy organisations, university medical schools, specialist medical colleges
Action	Identify, engage and prepare individuals for supporting implementation and overcoming resistance to clinical practice change.
Action target	General paediatricians and GPs
Temporality and dose	Not applicable

## 4 Discussion

We co‐developed strategies to address barriers and facilitators to the implementation of a paediatric risk‐reduction pathway for FH in Australia. FH is being detected increasingly across both tertiary lipid clinics and by GPs in primary care settings, creating blurred lines of responsibility for initiation of treatment and ongoing management. This awareness creates opportunities to identify and prepare paediatrician and GP champions, while empowering FH patients and families to engage in healthcare decisions. Nine potential implementation strategies were described to support a paediatric risk‐reduction pathway for FH, including (1) treatment guidance letter; (2) coordination and phone support; (3) individualised patient passport; (4) financial reimbursement for managing FH; (5) resources for diet and lifestyle; (6) FH patient and family engagement; (7) decision support systems; (8) audit and feedback quality assurance cycle; and (9) identifying and preparing clinical champions.

Previous research has described barriers and facilitators to the implementation of a paediatric risk‐reduction pathway [11]. From a patient perspective, lack of access to services and the cost of therapies has been an issue internationally [20]. Compared with the United States, the costs of statins are substantially cheaper through the Australian Pharmaceutical Benefits Scheme although higher than the United Kingdom [21]. The lack of access to dietitian advice and programs for FH in Australia is reflected internationally, where patients have described frustration at not receiving dietary advice as part of routine care [22]. Statin medication nonadherence is reported as a common barrier [22] but insights into children's perspectives of statin medication non‐adherence remain limited. Childhood adversity is thought to play a role, including conflicts, fear of a relative, illness, and financial strain [23]. The general lack of awareness and knowledge amongst health professionals in our study remains a prominent barrier to guideline implementation [22].

Our implementation strategies to support a paediatric risk‐reduction pathway for FH provide a novel contribution, as they were described with sufficient specificity to enable replication and scaling across settings. This approach can be adapted to other contexts, and its evaluation could build evidence for effective implementation in FH management. Previous research has taken a similar approach to the development of implementation strategies to improve treatment approaches for FH [18]. The creation of new clinical teams within a multidisciplinary lipid clinic has been identified as an effective approach to increased prescription of recommended treatment and reductions in LDL‐cholesterol levels [24]. The scalability and sustainability of centralised clinics to diagnose and treat up to 20 000 Australian children with FH remains questionable, particularly when community paediatrics and general practice can be harnessed through a shared‐care model.

Our study is limited by only considering group‐based deliberations through two focus group workshops. We did not undertake individual interviews which could have identified divergent opinions from what we uncovered in the group discussions. Despite our best efforts, the mix of stakeholders in each workshop was not always fully representative. Our focus on state‐wide and national issues limited the ability to delve into how this can be operationalised in individual practices or Primary Health Networks and the specifics of individual organisational challenges, such as how to best manage transition from paediatric to adult services. Guidance regarding transitional care is available in national and international guidelines [7, 8]. Our study had limited direct input from children or adolescents with lived experience of FH. Future research should focus on the operationalisation and evaluation of implementation strategies to support the adoption and sustainability of a paediatric risk‐reduction pathway in routine care.

## 5 Conclusion

We demonstrate the co‐design of a contextually grounded approach to developing implementation strategies for a paediatric FH risk‐reduction pathway in Australia. By engaging a broad range of stakeholders across state and national levels, we identified shared priorities, clarified optimal roles across care settings, and developed a suite of nine actionable strategies with sufficient specificity to support replication and adaptation. Our findings highlight the opportunity to leverage the capacity of community paediatrics and general practice within a shared‐care framework for the management of FH. This approach offers a replicable model for other jurisdictions aiming to improve the detection and management of FH in children, which could also apply to other major paediatric lipid disorders. Future research should focus on interventional studies of behaviour change for implementation practice and real‐world evaluation of these strategies to build a stronger evidence base for sustainable, system‐wide improvement in paediatric FH care.

## Funding

We would like to acknowledge that FH in Kids was funded by the Perth Children's Hospital Foundation and Telethon. Dr. Mitchell Sarkies is funded by an National Health and Medical Research Council (NHMRC) Investigator Grant (2007970) and Sydney Horizon Fellowship.

## Conflicts of Interest

Mitchell Sarkies, Shubha Srinivasan, Ari Horton, Kristen Nowak, Jing Pang and Andrew Martin: These authors declare no conflicts of interest. Jacquie Garton‐Smith: Accommodation and Travel to 2024 FH Summit partially funded by the Australian Atherosclerosis Society. Gerald Watts: Research grants and/or honoraria for lectures and/or advisory boards from Amgen, Arrowhead, CSL Sequirus, Esperion, Novartis, Novo Nordisk, Sanofi, Silence Therapeutics. Christine Tawtel: Novartis for events, OPHG for marketing. Jenny Della‐Vedova: Novartis for events, OPHG for marketing, accommodation and travel to 2024 FH Summit partially funded by the Australian Atherosclerosis Society.

## Supporting information


**Appendix S1:** Researcher characteristics. Description of the workshop facilitators' characteristics, including their occupation and experience.

**Appendix S2:** Facilitator guides. A summarised facilitator guide for the Perth workshop and National FH Summit workshop, including focus group questions.

**Appendix S3:** Standards for Reporting Qualitative Research Checklist. Table for the Standards for Reporting Qualitative Research (SPQR) Checklist.

## Data Availability

The data that support the findings of this study are available on request from the corresponding author. The data are not publicly available due to privacy or ethical restrictions.
