# Supplementary material for: Co‐Design of Strategies to Support the Implementation of a Paediatric Risk‐Reduction Pathway for Familial Hypercholesterolaemia
Source: J Paediatr Child Health. 2026 May 31;62(8):1411–20. doi: 10.1111/jpc.70447 (PMC13480714; doi:10.1111/jpc.70447)
Supplement: Supplementary file 1 [file JPC-62-1411-s001.docx]

**Supplementary Appendix 1. Researcher Characteristics**

| **Researcher** | **Characteristics** |
| --- | --- |
| Dr Mitchell Sarkies | Implementation scientist and physiotherapist with expertise in co-design and qualitative methods. His clinical background, doctoral training, and collaborations in cardiovascular care may have shaped interpretation; Dr Sarkies adopted a reflexive stance and prioritised participant-led contributions during facilitation and analysis. |
| Dr Andrew Martin | General paediatrician at Perth Children’s Hospital and academic at the University of Western Australia. He has led national efforts in paediatric FH, including authorship of the first Australian guidelines (2012) and establishment of a specialist clinical service in Western Australia. Dr Martin’s extensive clinical and research expertise in paediatric FH may have shaped interpretation; this was balanced by a reflexive approach and an emphasis on participant-led input during the workshops. |
