# Supplementary material for: Co‐Design of Strategies to Support the Implementation of a Paediatric Risk‐Reduction Pathway for Familial Hypercholesterolaemia
Source: J Paediatr Child Health. 2026 May 31;62(8):1411–20. doi: 10.1111/jpc.70447 (PMC13480714; doi:10.1111/jpc.70447)
Supplement: Supplementary file 2 [file JPC-62-1411-s002.docx]

**Supplementary Appendix 3. Standards for Reporting Qualitative Research Checklist**

| **Item** | **Page Number** |
| --- | --- |
| **Title & abstract** | |
| Title | 1 |
| Abstract | 1 |
| **Introduction** | |
| Problem formulation | 2 |
| Purpose of research | 2 |
| **Methods** | |
| Qualitative approach & research paradigm | 2 |
| Researcher characteristics & reflexivity | A3: 1 |
| Context | 2-3 |
| Sampling strategy | 3 |
| Ethical issues pertaining to human subjects | 3 |
| Data collection methods | 2 |
| Data collection instruments & technologies | 2 |
| Units of study | 3 |
| Data processing | 3 |
| Data analysis | 3 |
| Techniques to enhance trustworthiness | 3 |
| **Results/findings** | |
| Synthesis & interpretation | 3-5 |
| Links to empirical data | 4-5 |
| **Discussion** | |
| Integration with prior work, implications, transferability, and contribution(s) to the field | 6-7 |
| Limitations | 6-7 |
| **Other** | |
| Conflicts of interest | A1: 1-2 |
| Funding | A1: 2 |
