# Supplementary material for: Co‐Design of Strategies to Support the Implementation of a Paediatric Risk‐Reduction Pathway for Familial Hypercholesterolaemia
Source: J Paediatr Child Health. 2026 May 31;62(8):1411–20. doi: 10.1111/jpc.70447 (PMC13480714; doi:10.1111/jpc.70447)
Supplement: Supplementary file 3 [file JPC-62-1411-s003.docx]

**Supplementary Appendix 2. Facilitator Guides**

**Perth workshop focus group questions**

1. Who needs to do what at each stage?
2. Which patients are seen by who?
3. How do patients feel?
4. What support is needed for that to take place (Actor? Action? Target? When? How much?)
   a. Evaluation: Are we ready for change?
   b. Tailoring to local setting: What adaptations are needed to make this happen on the ground?
   c. Training & education: What training and education is needed?
   d. Engage consumers: How can patients be best engaged?
   e. Infrastructure: Any changes to IT systems?
   f. Facilitation & assistance: Role of clinic nurses to facilitate and assist?
   g. Stakeholder relationships: Contact between GPs & paediatricians & other clinicians?
   h. Support clinicians: Reminders, recalls, etc.?
   i. Funding: How do we make financially viable? (e.g. case conference MBS Item, chronic disease management plans, activity-based funding for hospital, etc.?

**National FH Summit workshop focus group questions**

1. What is getting in the way of implementing a shared care risk reduction pathway for kids with FH in your State/Territory?
2. What is currently working well for kids with FH in your State/Territory?
3. What needs to change in your State/Territory?
4. How do we make it happen?
   a. Are we ready for change?
   b. What adaptations are needed to make things happen on the ground?
   c. What training and education is needed?
   d. How can patients and families be best engaged?
   e. Do we need any changes to infrastructure (e.g. IT systems)?
   f. What is the role of nursing and allied health staff to facilitate and assist?
   g. Can we improve contact between GPs & paediatricians & other clinicians?
   h. What would be useful to support clinicians (e.g. reminders, recalls)?
   i. How do we make it financially viable? (e.g. case conference MBS Item, chronic disease management plans, activity-based funding for hospital?
